# Positive Affect and Cognitive Restoration: Investigating the Role of Valence and Arousal

**DOI:** 10.1371/journal.pone.0147275

**Published:** 2016-01-19

**Authors:** Logan J. Nealis, Zack M. van Allen, John M. Zelenski

**Affiliations:** 1 Department of Psychology and Neuroscience, Dalhousie University, Halifax, Nova Scotia, Canada; 2 Department of Psychology, Carleton University, Ottawa, Ontario, Canada; Radboud University Nijmegen, NETHERLANDS

## Abstract

Positive moods are thought to restore self-control resources following depletion. However, it is not well understood whether this effect is due to affective valence (pleasantness), arousal (activation), or a combination of both. Across four studies, we set out to investigate the role of positive moods on cognitive and behavioral measures of self-regulation in an ego-depletion paradigm. In studies 1 and 2, we independently manipulated affective valence and arousal and assessed self-regulation with a Stroop task. Results did not suggest a restorative effect of either on cognitive resources. In study 3, we employed both behavioral (the ‘handgrip task’) and cognitive (Stroop) assessments of self-regulation. Again, no significant effect of mood was observed on the Stroop task. Additionally, participants did not persist significantly longer on the handgrip task following a positive mood induction. Finally, in study 4, high vs. low states of arousal were manipulated and self-regulation was assessed via pre- and post-manipulation Stroop performance. In study 4, Stroop performance improved slightly more across time points for those in the high arousal condition than for those in the low arousal condition. Therefore, across four studies, we failed to find a consistent pattern of results suggesting that positive moods restore cognitive resources.

## Introduction

The ability to successfully self-regulate one’s thoughts, feelings, and impulses contributes to positive life outcomes such as having successful relationships, achieving academic and career success, and maintaining psychological and physical health. In contrast, self-regulatory failure is associated with increased drug use, higher rates of criminal convictions, and physical health problems [1]. Given the implications of self-regulatory success and failure in various life domains, it is important to determine the factors that facilitate successful self-regulation.

A series of studies conducted by Tice and colleagues [2] suggest that experiencing positive emotions may help people self-regulate more effectively when their self-control might otherwise fail [2]. However, these studies were not optimally suited to determine whether this effect was due the *valence* (i.e., pleasantness) or *arousal* (i.e., activation) of the positive emotions. Here we report on four studies that examine the relationship between mood and self-regulation by manipulating moods’ valance and arousal, and testing effects on cognitive and behavioral control.

### Valence versus arousal

In contrast to conceptualizing emotions as discrete entities [3], researchers often consider emotions, and affective states more broadly, from a dimensional perspective. With this approach, feeling states are organized according to co-occurrence or experiential similarity. Different feelings fit within a two-dimensional space, referred to as the *affect circumplex* [4]. Commonly—and in the paper by Tice et al. [2] and the current investigation—these dimensions of affect are defined as valence and arousal.

In the affect circumplex, positive moods may be either high or low arousal [4]. For example, joy and excitement are positive, high arousal moods, whereas contentment and relaxation are positive, low arousal moods. Popular measures of affect, such as the Positive and Negative Affect Schedule (PANAS) [5], often focus on activated pleasant states. Absent careful attention to assessment or manipulation of lower arousal pleasant states, the effects of positive moods on self-regulation could conceivably result from the valence of the mood, the arousal of the mood, or both.

### Ego-Depletion & Affect

“Ego-depletion” refers to the state of mental fatigue that results from the exertion of self-control [6]. Of the various models proposed to explain the depletion effect, the strength model has been subject to the most empirical investigation. The results of numerous studies are consistent with this model; however, there is also ongoing debate about the extent to which strong depletion effects may be attributable to publication bias (e.g., [7][8]). According to the limited strength model of self-control, self-regulation functions analogous to a muscle in that it becomes fatigued with use, is restored naturally with rest, and is strengthened with repeated use [9]. Accordingly, depletion is thought to affect a shared resource, such that the depletion of this resource can diminish self-regulation of behavioral inhibition, emotional regulation, and cognitive control. Thus, inhibiting a behavior can subsequently hinder attempts to regulate emotion or perform complex cognitive tasks, and vice versa [9] [10]. Automatic processes, however, do not share this common resource and thus do not contribute to, and are not affected by, states of resource depletion [11].

Factors that facilitate self-control when mental resources are depleted include being motivated to perform the task [12] and affirming one’s core values [13]. Additionally, Tice, Baumeister, Schmeuli & Muraven [2] found that experiencing positive emotions facilitates self-regulatory success following earlier depletion.

Across four studies by Tice et al. [2], experiencing positive moods after being depleted had a restorative effect on people’s ability to self-regulate. Participants were initially depleted using various strategies, including thought suppression, behavioral inhibition, and habit-breaking tasks. Participants were then put into positive, sad, or neutral moods with video clips or through receiving either a surprise gift (positive) or a paper receipt (neutral). Available mental resources were then measured with task persistence or muscular endurance. The researchers found that people who experienced positive moods after their mental energy was depleted were able to perform tasks with the same degree of efficiency as people who had not been depleted. Those who were depleted and experienced either neutral or negative emotions had poorer performance on whatever measurement task was used in the given study [2]. Although these effects appeared uniformly across these four studies, the effect of pleasant moods may be confounded, at least in part, by other aspects of affect (e.g., arousal).

In their third study, Tice et al. [2] address this issue directly. Because they used a mood scale that contained both a valence and an arousal subscale, the authors separated valence and arousal statistically to test which dimensions independently predicted their outcome measure (persistence on a hand-grip task). The authors concluded that the valence of the mood, rather than the arousal, was responsible for increased performance on the hand-grip task [2]. However, a single test with a small sample size (N = 42) is not conclusive. A more powerful strategy would be to independently manipulate arousal and valence (i.e., rather than merely assess them), and this is the approach we have taken.

Instead of bringing clarity to the issue, other research suggests an uncertain role of arousal in restoring self-regulatory resources. High-arousal states are thought to interfere with performance on the classic Stroop task—used to assess depletion because it requires inhibition—while low-arousal states facilitate performance [14]. However, in one ego-depletion study, people in a depleted state reported a higher incidence of subjective fatigue or tiredness than those who were not depleted [9]. Although no causal relationship is identified, it suggests that low arousal coincides with states of depletion. In sum, the role of arousal in restoration is complex and not well understood. Further research with improved methodology should help clarify the issue.

### Current studies

The present research was designed to further explore the restorative effect of positive emotions on self-regulatory resources by extending the results to different outcome measures and offering improved methodology to clarify the role of valence and arousal in this process. Across four studies, we use an ego-depletion paradigm to investigate the effects of affective valence and arousal on cognitive control.

In study 1, we used a lab-based mood induction (music & images), and examined the effect on response-inhibition via performance on a Stroop task [15]. In study 2, we used a more experiential mood induction (walking vs. sitting in pleasant vs. neutral surroundings) along with the same depletion and measurement tasks as in study 1. Studies 1 and 2 did not provide strong support that affective valence or arousal produce restorative effects on cognitive control. Thus, in study 3 we employed an experimental approach more closely resembling the design and handgrip measure employed by Tice et al [2] in an attempt to replicate the original findings. In study 4, we focused on manipulating arousal (high vs. low) via physical exertion (exercise vs. sitting) and examined the effect on Stroop performance.

## Study 1

We made two hypotheses for study 1. First, according to ego-depletion theory and research (i.e., a shared resource exists and is restored by pleasant moods), we hypothesized that those people who experience pleasant moods after being depleted will show improved performance on a cognitive control task relative to those people who experience neutral moods. Second, in line with hints from previous research [2] [14], we did not expect that arousal would improve performance.

### Methods

#### Participants

Participants were recruited from a university participant pool consisting of undergraduate students enrolled in first and second year psychology classes. Participants received course credit for their participation. The Carleton University Ethics Committee for Psychological Research approved this experiment. Data was collected from 131 students, and 22 were excluded from analysis for not following task instructions (*n* = 20), or disclosing color-blindness (*n* = 1) or an attention disorder (*n* = 1). Analysis included 109 participants (50 = male, 50 = female, 9 = unspecified), between the ages of 17 and 50 (*M* = 21.07, *SD* = 5.18). Twenty-seven participants (25.7%) reported a preference for classical music, and 30 participants (28.4%) reported having formal musical training (*M* = 5.39 years, *SD* = 3.6 years). All participants provided written consent to participate in the study.

#### Procedure

After giving informed consent, participants completed a short package of demographic and personality questionnaires. Participants then completed a habit-breaking task to deplete their self-regulatory resources. Following this task, the Stroop task [15] was administered to each participant to obtain a baseline for speed and accuracy while in a depleted state. Participants were randomly assigned to one of four mood conditions: positive high-arousal, positive low-arousal, neutral high-arousal, and neutral low-arousal. Participants were randomly assigned to watch one of the 5-minute video clips to put them into one of the desired mood states. After the video, participants completed a measure of their mood state. The Stroop task was administered to participants a second time to measure cognitive performance following the mood induction. Participants were then given verbal and written debriefing, with a probe for suspicion regarding the true intentions of the study.

### Materials

#### Background information and personality

Participants completed a short self-report demographic questionnaire, which included questions relating to their gender, age, year of study, program of study, music preferences, and music training. Additional questionnaires assessing personality variables were also collected, but were not included in analysis.

#### State mood

Participants’ state mood was measured with a 27-item questionnaire with mood adjectives from the affect circumplex [4]. This included adjectives related to arousal (*stimulated*, *full of energy*, *quiet*, *tired*) and valence (*happy*, *pleased*, *unhappy*, *sad*). Other adjectives, similar to those used in the Positive and Negative Affect Schedule (PANAS; [16]), were included but not used in analysis due to their composition of both valence and arousal dimensions. Participants were asked to, “indicate to what extent [they] feel this way right now, at this moment” on a 7-point Likert Scale from *very slightly or not at all* (1) to *extremely or a lot* (7) for each of the adjectives. Valence was calculated by averaging the two pure positive valence items (*happy*, *pleased*) and the reverse-scored pure negative valence items (*sad*, *unhappy*). Arousal was calculated by averaging the two pure high arousal items (*full of energy*, *stimulated*) and the reverse-scored pure low-arousal items (*quiet*, *tired*). Internal consistency was acceptable for both valence (*α* = .78) and arousal (*α* = .69).

#### Depletion task

Self-regulatory resources were depleted using a habit-breaking task. In this task, participants were first asked to read a book excerpt about the history of political thought (454 words; [17]) and cross out every letter ‘e’ that they found so that a habit was created. Participants were then asked to read a second excerpt from the same book (391 words) and cross out every letter ‘e’ that they saw, except if the letter ‘e’ was beside a vowel, or one letter away from a vowel. This forced participants to override their previously learned habit. This task was among the most widely used, effective, and consistent depletion techniques evaluated in a meta-analysis [18] and was used in two of the four restoration studies that motivated this research [2].

#### Mood induction

Mood was induced using a combination of images from the International Affective Picture System (IAPS; [19]), and instrumental music recordings. The images and music were compiled into five-minute long videos. Each video was composed of 50 unique images (each displayed for 5 seconds), with a 1 second fade between each image. A 3 second fade was applied to the music at the beginning and end of the videos to avoid abrupt changes in volume. Participants were instructed to watch the videos and pay attention to the images and music. Mood conditions varied orthogonally by valence (positive, neutral) and arousal (high, low), which created four mood conditions: (positive high-arousal, positive low-arousal, neutral high-arousal, neutral low-arousal).

The positive high-arousal condition contained images with positive and active themes (e.g., victorious athletes, children on waterslides, etc.) along with music from *Slavonic Dance No*. *5 in A* Major by Antonin Dvorak [20]. The positive low-arousal condition contained images with positive but relaxing themes (e.g., smiling faces, landscapes, etc.) along with music from *Venus*, *the Bringer of Peace* by Gustav Holst [21]. The neutral high-arousal condition contained images with active but neither pleasant nor unpleasant themes (e.g., an emergency room, busy urban environments, etc.) along with music from *Mars*, *the Bringer of War* by Gustav Holst [21]. The neutral low-arousal condition contained images that were not active, and neither pleasant nor unpleasant (e.g., neutral faces, household objects) along with music from *Adagio for Strings* by Samuel Barber [22].

IAPS photos were chosen according to their published valence and arousal ratings [19], and musical selections were chosen according to previous research [23] [24]. The combination of IAPS photos and instrumental music has been used successfully in previous research as an independent manipulation of valence and arousal [25], and a pilot study verified that the mood induction had the desired effect on mood. The 200 IAPS photos used in the experimental manipulation are listed online with the publicly available datasets.

#### Cognitive control task

Cognitive control was measured using a computer-administered Stroop task [15]. This involved presenting participants with color names (red, green, blue, yellow, and purple) that were printed in text that was either color-congruent (e.g., the word ‘red’ printed in red text) or color-incongruent (e.g., the word ‘red’ printed in green text). Participants were asked to identify the color of the text, not the word, for each trial using a special keypad with a button for each color (from left to right: red, yellow, green, blue, and purple). Participants were instructed to respond as quickly and accurately as possible during the task. Research has validated the Stroop task as a measure of cognitive control in self-regulation research [18] [26].

The task consisted of 325 trials in total. Of these, 65 trials (20%) were color-congruent, and 260 (80%) were color-incongruent. These were randomly presented in 13 experimental blocks with 25 trials in each block. In each block, each possible combination of color name and color text (including color-congruent and color-incongruent trials) was presented once in random order. This task took approximately 5 minutes to complete.

Stroop data was cleaned and processed in R [27] and was done consistently across the four studies. Visual inspection of data indicated observable practice effects in the initial blocks of the Stroop task, so the first 50 trials were considered “practice” and were not retained in subsequent analyses. In studies involving pre- and post-induction Stroop tasks (i.e., study 1 and study 4), practice effects were observable only in the first Stroop task, and thus all 325 trials were retained in post-Stroop tasks. We examined an alternative trimming strategy where the first 50 trials were removed from both tasks in studies 1 and 4 (to keep this constant); scores were correlated *r* > .95 across criteria, and no results differed meaningfully with alternative scores.

To account for accidental key presses and lapses in attention, only response times between 100 ms and 5000 ms were considered true responses, and values outside this range were classified as missing. Response times, in milliseconds, were then log transformed to better approximate a normal distribution and outliers were identified within each participant separately. Responses greater than three standard deviations from the individual’s mean were classified as missing. Across all four studies, 98.4% of trials were retained for analysis. Trimmed data were used to calculate mean log response times, incongruent RTs (RTs from incongruent trials), interference scores (calculated by subtracting congruent from incongruent RTs), accuracy scores (percentage of correct trials), and accuracy interference scores (calculated by subtracting congruent from incongruent accuracy). All calculations are available in the accompanying data set; however, only mean log RTs are reported in this manuscript as analyses with these alternative scoring schemes did not produce any consistent pattern of divergent results.

### Results

#### Preliminary Analyses

Because people’s musical preferences and past musical training might influence the efficacy of the mood induction, both of these factors were investigated. There were no significant differences by condition among people who reported a preference for classical music, *χ*^*2*^ (3, *N* = 109) = .75, *p* = .86, and of people who reported formal training in music, *χ*^*2*^ (3, *N* = 109) = 1.91, *p* = .59. Neither preference for classical music nor past musical training influenced the results. Because frequencies were not different between mood conditions and the two variables were unrelated to our dependent measures, they were not included as covariates in further analyses.

#### Manipulation check

To determine whether the mood induction was successful at putting people into the desired moods, we conducted two separate ANOVAs: one on self-reported valence, and one on self-reported arousal. A 2 (valence) x 2 (arousal) between-subjects ANOVA was conducted on self-reported valence. There was a significant main effect of valence in the valence condition, *F* (1, 105) = 14.99, *p* < .001, partial *η*^*2*^ = .12, but no significant main effect of arousal in the valence condition, *F* (1, 105) = 3.33, *p* = .07, partial *η*^*2*^ = .03. No significant valence x arousal interaction was present, *F* (1, 105) = 0.55, *p* = .46, partial *η*^*2*^ = .01. Thus those watching the pleasant videos reported more pleasant emotions (*M* = 5.12, *SD* = 1.15) than those watching the neutral videos (*M* = 4.10, *SD* = 1.29). In contrast, those who watched the high-arousal videos (*M* = 5.35, *SD* = 1.22) did not report significantly more pleasant emotions than those who watched the low-arousal videos (*M* = 4.67, *SD* = .87).

A 2 (valence) x 2 (arousal) between-subjects ANOVA was also conducted on self-reported arousal. There was no main effect for valence conditions on self-reported arousal, *F* (1, 105) < .01, *p* = .95, partial *η*^*2*^ = .00, but a main effect was found for arousal conditions on self-reported arousal, *F* (1, 105) = 15.89, *p* < .001, partial *η*^*2*^ = .13. No valence x arousal interaction was found, *F* (1, 105) = .64, *p* = .43, partial *η*^*2*^ = .01. This shows that those who watched the high arousal videos reported greater arousal (*M* = 3.97, *SD* = 1.04) than those who watched the low arousal videos (*M* = 2.88, *SD* = 1.03), while those who watched the pleasant videos reported a similar amount of arousal (*M* = 3.81, *SD* = 1.50) as those who watched the neutral videos (*M* = 3.07, *SD* = 1.17).

#### Effect of Mood on Cognitive Performance

A 2 (valence) x 2 (arousal) x 2 (time) mixed-factors ANOVA was conducted on mean Stroop response times. A significant main effect of time on Stroop response time was found across the two time points indicating that, on average, response times decreased across time points, *F* (1, 105) = 389.48, *p* < .001, partial η^2^ = .79, but no significant main effect was found for either valence, *F* (1,105) = 1.03, *p* = .31, partial η^2^ = .01, or arousal conditions, *F* (1,105) = .63, *p* = .43, partial η^2^ = .01. A marginally significant valence x time interaction was found, *F* (1,105) = 3.73, *p* = .06, partial η^2^ = .03, indicating the decrease in response times across time points was greater for neutral valence conditions than positive valence conditions. This interaction pattern was inconsistent with hypotheses. No other interactions were found to be significant, including valence x arousal, *F* (1,105) = 1.50, *p* = .22, partial η^2^ = .01, arousal x time, *F* (1, 105) = 3.22, *p* = .08, partial η^2^ = .03 or valence x arousal x time, *F* (1, 105) = 2.66, *p* = .11, partial η^2^ = .03. Mean log Stroop RTs for study 1are presented in Table 1.

**Table 1:** Mean log Stroop response times (RTs) in Study 1.

		Pre		Post	
Arousal	Valence	M	SD	M	SD
Low	Neutral	2.967	.063	2.908	.053
	Positive	2.934	.063	2.891	.063
High	Neutral	2.937	.062	2.894	.056
	Positive	2.938	.060	2.897	.056

### Discussion

This investigation extends research on the potential restorative function of positive moods on depleted self-regulatory resources. Particularly, we made methodological improvements (e.g., independent manipulation of valence and arousal) to better understand which dimensions of mood might be responsible for this effect (if it exists). To accomplish this, we depleted participants’ resources, measured their cognitive control, induced moods that varied independently by valence and arousal, and re-measured their cognitive control. We expected that people in the positive valence mood conditions would show an improvement of cognitive control relative to those in neutral valence mood conditions. In line with previous research, we expected that differences in arousal would play a minor role in restoration.

Surprisingly, despite successful mood inductions, we failed to find a unique restorative effect of either valence or arousal on self-regulatory resources. We observed a substantial decrease in response time when comparing performance before and after the mood induction, indicating that restoration did occur. This effect, however, did not differ significantly based on valence, arousal, or a combination of the two dimensions. A marginally significant valence by time interaction was observed; however, the pattern suggested that restoration was facilitated more by neutral valence than positive valence. Whether we failed to find an effect of positive valence due to our method of mood induction or type of self-regulatory resources is ambiguous. In light of these results, we decided to conduct a follow-up study using a different method of mood induction to better approximate mood states as experienced on a daily basis.

## Study 2

In study 2, we modified some of the elements from study 1 while retaining its core features. In this study, we conducted an experiential mood induction outside of the lab (walking vs. sitting in pleasant vs. neutral surroundings) and compared differences in post-induction Stroop performance between groups. Our hypotheses were the same as those in study 1.

### Method

#### Participants

Participants were again recruited from a university participant pool and were awarded course credit for their participation. A total of 159 participants were tested with 43 excluded from analysis. This was due to rainy weather on the day of the experiment (*n* = 29, which meant we could not randomly assign to the outdoor conditions), for not following instructions (*n* = 4), or for not completing the study in a reasonable timeframe (*n* = 10). Analysis included 116 participants (44 male, 66 female, 6 unspecified) between the ages of 17 and 37 years (*M* = 19.7, *SD* = 2.83). This study was approved by the Carleton University Ethics Committee for Psychological Research. Participants provided written consent to participate in the study.

### Materials

#### Background information and personality

Participants completed self-report questionnaires about background and personality information similar to that in study 1.

#### State mood

State mood was measured before and after the mood induction using a 24-item version of the mood questionnaire [16]. Because there is some debate about how closely *tired* corresponds to pure low-arousal [28], this adjective was replaced with *idle* to better capture a state of inactivity. This modification did not substantially alter the inter-item reliability of the scale (valence *α* = .73; arousal *α* = .61).

#### Depletion task

The habit-breaking task used in study 1 was again used as a method of depleting self-regulatory resources.

#### Mood induction

Mood was induced between-subjects so that participants differed in both valence (positive vs. neutral) and arousal (high vs. low). This resulted in four distinct moods: positive-high arousal, positive-low arousal, neutral-high arousal, and neutral-low arousal. Valence was manipulated by varying the environment that participants were exposed to, including natural outdoor environments around campus (positive valence) and artificial indoor environments in the campus tunnel system (neutral valence; cf., [29]). Physical activity was varied during this exposure to create high arousal (brisk walking) and low arousal (sitting) conditions (cf., [30]). Each induction lasted approximately 12 minutes. Walking routes were matched for total distance, and sitting locations were matched for distance from the lab. Walking speed was kept constant throughout the induction and between sessions.

#### Cognitive control task

The same Stroop task used in study 1 was again used to measure cognitive control.

#### Procedure

Participants arrived at the lab ostensibly for a study about the effect of campus environments on mental functioning. Participants were run in groups of between one and three people. Each group was randomly assigned to an experimental condition (positive-high arousal, positive-low arousal, neutral-high arousal, neutral-low arousal) before the session began. Each participant was seated in a separate room. After obtaining informed consent, participants completed questionnaires relating to background information and personality. Participants were then given the depletion (habit breaking) task to deplete their mental resources, which took approximately 15 minutes to complete. Participants completed a questionnaire to measure their state mood at that moment.

The experimental groups then received the mood induction procedure according to their assigned condition. In both conditions participants walked approximately 600 meters. In each condition, participants were asked to observe and be attentive to their environment and to keep interactions among themselves and with others to a minimum. Back at the lab, participants completed a post-induction measure of mood to assess how they had felt during the manipulation. In separate rooms, participants completed the Stroop task to measure their level of cognitive control. Participants were debriefed with a probe for suspicion regarding the true intentions of the study.

### Results

#### Manipulation Check for Mood

As with study 1, we sought to confirm whether the mood induction procedure was successful at inducing the desired moods. We again conducted two separate ANOVAs: one on self-reported valence, and one on self-reported arousal. A 2 (valence) x 2 (arousal) between-subjects ANOVA was conducted on self-reported valence. There was a significant main effect of valence condition on self-reported valence, *F* (1, 112) = 19.34, *p* < .001, partial η^2^ = .15, but no main effect of arousal condition on self-reported valence, *F* (1, 112) = 0.71, *p* = .40, partial η^2^ = .01. No significant valence x arousal interaction was found, *F* (1, 112) = 0.01, *p* = .92, partial η^2^ = .00. Thus those in pleasant valence conditions reported more pleasant emotions than those in neutral valence conditions. In contrast, those in the high arousal conditions reported similar levels of pleasant emotions as those who in the low arousal conditions.

A 2 (environment) x 2 (activity level) between-subjects ANOVA was also conducted on self-reported arousal. There was a significant main effect for arousal condition on arousal, *F* (1,112) = 55.70, *p* < .001, partial η^2^ = .33. There was also a small but significant main effect of valence condition on arousal, *F* (1,112) = 6.19, *p* = .01, partial η^2^ = .05. No valence x arousal level interaction was found, *F* (1, 112) = .24, *p* = .63. partial η^2^ = .00. This shows that those in the high arousal conditions reported greater arousal than those in the low arousal conditions. Additionally, people in the pleasant valence conditions also reported higher arousal than people in the neutral valence conditions. Although the valence and arousal conditions both appear to affect arousal, the relatively small effect size for valence condition in this case suggests that the mood induction generally put people into the desired moods.

#### Effect of Mood on Cognitive Performance

A 2 (valence) x 2 (arousal) ANOVA was conducted on mean Stroop response times. Main effects were not significant for either valence, *F* (1, 112) = 1.01, *p* = .38, partial η^2^ = .01, or arousal *F* (1, 112) = .16, *p* = .69, partial η^2^ = .00. No valence x arousal interaction was found *F* (1, 112) = .85, *p* = .36, partial η^2^ = .01. Mean Stroop RTs are presented in Table 2.

**Table 2:** Mean log Stroop response times (RTs) in Study 2.

Arousal	Valence	M	SD
Low	Neutral	2.930	.046
	Positive	2.930	.055
High	Neutral	2.915	.055
	Positive	2.936	.073

### Discussion

In study 2 we manipulated affective valence and arousal by varying activity levels and physical environments and assessed cognitive control with performance on a Stroop task. Although differences in the environment manipulation showed effects on valence only, the activity level showed effects on both valence and arousal. It should be noted, however, that the effect of experiencing different levels of activity on arousal was much larger than the effect on valence. Thus, we are confident that participants across the two studies were experiencing moods close to those that we intended.

Despite generally successful mood inductions, we failed to find any support for our hypotheses that post mood induction Stroop performance would differ by affective valence or arousal. Following this second set of null results we speculated that the discrepancy between our findings and those by Tice et al [2] may be due to our choice in self-regulation assessments. Tice et al [2] utilized behavioral measures of self-regulation (drinking unpleasant beverages, persistence on a frustrating or unsolvable task, and physical stamina as measured by the hand grip task) while we employed a cognitive measure of self-regulation (the Stroop task). Therefore, we decided to conduct a third study that employed both cognitive and behavioral assessments of self-regulation.

## Study 3

Following unexpected results in studies 1 and 2 we sought to replicate the finding that positive emotion can restore cognitive resources using a similar methodology to that used by Tice et al., [2]. In study 3 we did not independently manipulate valence and arousal but instead manipulated positive mood with a video clip and assessed ego-depletion with both a measure of physical stamina (the ‘handgrip task’) and the Stroop task.

### Method

#### Participants

Seventy-seven participants were recruited from a university participant pool and were awarded course credit for participating. Fifteen participants were excluded from analysis for procedural issues and failing to follow instructions. Analysis included 62 participants (45 women, 17 men) between the ages of 18 and 35 years old (*M* = 19.98, *SD* = 3.03). Participants were randomly assigned to a condition before arrival (positive *n* = 32, neutral *n* = 30). This study was approved by the Carleton University Ethics Committee for Psychological Research. Participants provided written consent to participate in the study.

#### Procedure

Participants provided written consent, completed demographic and personality questionnaires, and the same depletion task used in studies 1 and 2. A handgrip task was then administered to obtain a baseline measure of stamina while in a depleted state. Following random assignment to condition, participants viewed a ten-minute film clip meant to induce a positive or neutral mood. Participants then completed a brief affect questionnaire followed by a second handgrip task. Finally, participants completed a computer-based Stroop task.

### Materials

#### Background information and personality

Participants completed self-report questionnaires relating to background and personality information similar to those in Studies 1 and 2.

#### State mood

State mood was measured after the mood induction using a 28-item version of the mood questionnaire [16].

#### Handgrip task

The handgrip task measures the duration of time an individual can contract a handgrip (a hand exercise device comprised of two handles and a metal spring) sufficiently tight to hold a small object between the handles. The handgrip task is commonly used in the ego-depletion literature [2][9]. Sitting in a chair with their dominant arm on the armrest, participants contracted an Energetics brand handgrip tight enough to hold a 1.2 cm wide eraser between the handles. Using a stopwatch the experimenter timed the duration of time each participant could hold the eraser between the handles of the handgrip.

#### Mood induction

Mood was induced by one of two ten-minute long films previously demonstrated to induce either a positive or a neutral mood [31]. A scene from ‘E.T.: The Extra-Terrestrial’ that shows children successfully rescuing a young extra-terrestrial from the authorities [32] was used as the positive mood manipulation. A ten-minute clip from a documentary about a painter [33] was used as the neutral mood manipulation.

### Results

#### Manipulation Check

A series of independent samples *t*-tests were employed to assess the effectiveness of the mood induction manipulation. Self-reported valence and arousal were both greater in the positive condition. Specifically, those in the positive condition (*M* = 6.15, *SD* = .72) reported higher levels of affective valence than did those in the neutral mood condition (*M* = 5.32, *SD* = .89), *t*(60) = 4.05, *p* < .001, *d* = .*74*. However, participants in the positive condition (*M* = 4.56, *SD* = 1.20) also reported similarly more affective arousal than participants in the neutral mood condition (*M* = 3.28, *SD* = 1.10), *t*(60) = 4.38, *p* < .001, *d* = .80.

#### Handgrip

A 2 (Time) x 2 (Condition) mixed ANOVA was conducted on handgrip persistence. Main effects were not significant for Time, *F* (1, 60) = 3.29, *p* = .08, *η*^*2*^ = .05, or Condition, *F* (1, 60) = 2.714, *p* = .11, *η*^*2*^ = .04. The Time x Condition interaction was not significant, *F* (1, 60) = .227, *p* = .64, *η*^*2*^ = .00. Handgrip persistence times are presented in Table 3.

**Table 3:** Handgrip persistence times in seconds for Study 3.

	Pre		Post	
Valence	M	SD	M	SD
Neutral	26.37	23.49	22.03	18.42
Positive	34.84	26.92	32.31	24.47

Because some non-significant effects were of modest size (or ‘marginally significant’), we explored some follow-up tests. Repeated measure t-tests revealed that handgrip times in the neutral mood condition decreased from the first handgrip task (*M* = 26.37s, *SD* = 23.49s) to the second (*M* = 22.03s, *SD* = 18.42s), *t*(29) = 2.04, *p* = .05, *d* = .36, while there was no significant difference in handgrip times in the positive mood condition from the first task (*M* = 34.84s, *SD* = 26.92s) to the second (*M* = 32.31s, *SD* = 24.47s), *t*(31) = .82, *p* = .42, *d* = .*12*. Thus, the pattern of simple effects plausibly support a weak, relative restorative effect of positive moods.

#### Effect of Mood on Cognitive Performance

Independent t-tests were conducted to compare mean Stroop response times between conditions. The mean Stroop response times for those in the positive mood condition (M = 2.913, SD = .050) did not differ significantly from those in the neutral mood condition (M = 2.929, SD = .047), t(60) = 1.32, p = .19, d = .33.

### Discussion

In study 3 we sought to replicate the findings of Tice et al., [2] by manipulating positive affect and assessing ego-depletion with persistence on a handgrip task, in addition to the Stroop task. We intended to primarily manipulate affective valence, and in this regard the mood manipulation was successful. However, the manipulation also increased affective arousal. Thus, the positive mood manipulation produced high-arousal positive mood.

We observed that, in the neutral condition, participants persisted less on the handgrip task after watching the video clip than they did immediately following the depletion task. The observed effect in the neutral mood condition was small to moderate (*d* = .36; [34]) and on the threshold of conventional standards of statistical significance; however, it indicates that participants gave up on the task more quickly after the neutral mood induction, compared to immediately following depletion. In contrast, handgrip persistence did not differ significantly across time points in the positive mood condition. Thus, participants persisted on the handgrip task for approximately the same duration immediately following depletion as they did following the positive mood induction. In fact, handgrip persistence decreases over the two assessments (although the difference was not significant).

Because the neutral mood induction lead to a within-person decrease in handgrip persistence while the positive mood induction did not, it is possible to interpret these results as supporting a beneficial effect of positive mood on self-regulation. That is, it is plausible that the positive mood induction incubated participants from the detrimental effects observed in the neutral mood condition. However, the lack of within-person improvement in handgrip persistence from a depleted state to a positive mood state also suggests that participants’ self-regulatory abilities did not fully ‘restore’. Therefore, the results of this study do not provide strong evidence for the restorative function of positive mood on self-regulatory abilities.

## Study 4

In Studies 1 and 2 we manipulated valence and arousal independently. Although a more elegant approach compared to a single manipulation, those efforts were not rewarded with clear findings. In study 3 we targeted affective valence only as a pragmatic choice to reduce sample size, but the manipulation influenced arousal as well. We made a similar pragmatic choice in study 4 and opted to round out our investigation by focusing our affect manipulation on arousal. That is, we sought to isolate the potential role of arousal in the restorative process by manipulating states of arousal (high vs. low) and assessing within- and between-subjects differences in Stroop performance.

### Method

#### Participants

Sixty-two participants completed the study in exchange for course credit. Three participants were excluded from analysis for either not following instructions (*n* = 2) or for color blindness (*n* = 1). Analysis included 59 participants (18 male, 40 female, 1 unspecified) between the ages of 18 and 52 (*M* = 21.8, *SD* = 6.57). Participants were randomly assigned to either a low arousal (*n* = 35) or high arousal condition (*n* = 24). This study was approved by the Carleton University Ethics Committee for Psychological Research. Participants provided written consent to participate in the study.

#### Procedure

Participants enrolled in a study entitled “personality and cognition” in exchange for course credit. After completing a consent form, demographic questionnaire, and personality measure, participants finished the written ego-depletion task. Following the depletion task, a brief mood assessment was administered. Participants then completed the Stroop task in order to obtain a measure of cognitive control while in a depleted state. Next, participants were randomly assigned to the low or high arousal conditions. In the low arousal condition, participants were asked to sit in their chair and relax for 5 minutes. In the high arousal condition participants completed 4 sets of 30 jumping jacks with one-minute rest periods between each set (the average time for this task took 5 minutes). Following completion of the condition-specific activities, participants completed a mood measure and a second iteration of the Stroop task. Finally, participants were given a verbal and written debriefing.

### Materials

#### Background information and personality

Participants completed demographic and personality questionnaires similar to those used in studies 1–3.

#### State mood

State mood was assessed with the same measure employed in study 3.

#### Depletion task

Consistent with the previous studies self-regulatory resources were depleted by asking participants to form and break a habit in a short writing task.

#### Mood induction

Mood was induced between-subjects so that participants differed in their levels of arousal (high vs. low). Arousal manipulations were selected based on their demonstrated efficacy in previous research [35]. In the low arousal condition participants sat quietly in an empty room for five minutes. In the high arousal condition participants completed 4 sets of 30 jumping jacks with one-minute rest periods between each set. The task took approximately five minutes to complete.

#### Cognitive control task

A computer administered Stroop task was again used as a measure of cognitive control. The number of total, congruent, and incongruent trials were identical to those in studies 1–3.

### Results

#### Manipulation check for mood

Following the manipulation, self-reported arousal was significantly higher in the high arousal condition (*M* = 5.06, *SD* = 1.21) than in the low arousal condition (*M* = 3.74, *SD* = .98), *t*(57) = 4.61, *p* < .001, *d* = 1.22. Participants in the high arousal condition (*M* = 5.90, *SD* = .74) also reported slightly more positive affective valence than those in the low arousal condition (*M* = 5.43, *SD* = 1.15); however, this difference was not statistically significant, *t*(57) = 1.76, *p* = .08, *d* = .47.

#### Effects of mood condition on cognitive performance

A 2 (arousal) x 2 (time) mixed ANOVA was conducted on mean Stroop response times in order to determine whether differences in arousal affected cognitive control following depletion. A significant main effect was found across time points, *F* (1, 57) = 153.27, *p* < .001, partial η^2^ = .73, indicating that mean response times decreased over time; however, no main effect was found for the arousal condition, *F* (1, 57) = .01, *p* = .94, partial η^2^ = .00. An arousal condition x time interaction was found, *F* (1, 57) = 6.77, *p* = .01, partial η^2^ = .09 indicating that the performance increase on the Stroop task from pre-test to post-test was greater for the high arousal condition than the low arousal condition. Stroop RTs are presented in Table 4.

**Table 4:** Mean log Stroop response times (RTs) in Study 4.

	Pre		Post	
Arousal	M	SD	M	SD
Low	2.972	.057	2.937	.049
High	2.982	.073	2.929	.057

### Discussion

In study 4 we manipulated high and low states of arousal by varying levels of physical activity. Our manipulation created largely divergent states of arousal between conditions. The high arousal manipulation also seemed to increase levels of affective valence slightly, although the difference in valence was not statistically significant. Thus, the mood manipulation generally produced the intended effects.

As in study 1, we observed decreases in mean Stroop response times across time points, which could indicate practice effects, affect-linked cognitive restoration, or restoration due only to time. However, the presence of a time x arousal interaction suggests that the increase in performance across time points was greater in the high arousal condition than the low arousal condition. Although this effect was small, these results suggest that some restoration did occur, and that high levels of arousal enhanced this effect.

## General Discussion

Across four studies, with different mood inductions and both within- and between-persons comparisons, no consistent pattern of results emerged regarding the effect of affective valence or arousal on cognitive restoration in an ego-depletion paradigm. This contrasts with previous research indicating restorative effects on behavioral and cognitive inhibition tasks [2] [36].

Our mood inductions were relatively successful at independently manipulating the valence and arousal of mood. In study 1, differences in valence conditions caused differences in self-reported valence but not arousal. Likewise, differences in arousal conditions caused differences in self-reported arousal but not valence. In study 2, the valence manipulation (pleasant environment) produced effects only on affective valence whereas the arousal manipulation (physical activity) had an impact on both arousal and valence. In study 3 we intended to manipulate valence with a video-clip but instead elevated levels of both valence and arousal. Finally, in study 4 we successfully manipulated high vs. low states of arousal although some (statistically non-significant) differences we present in valence. In addition to manipulating valence and arousal relatively independently, our mood inductions created substantial differences in participants’ moods.

After carefully comparing and contrasting our studies with Tice et al.’s studies, the difference in dependent variables (i.e., cognitive vs. behavioral control) seems a plausible explanation for divergent restoration results in studies 1, 2, and 4. Although a plausible pattern of *relative* restoration could be extracted from the handgrip data in study 3, our results do not provide strong support for the restorative effect of positive mood on behavioral control. It remains plausible that more behavioral (vs. cognitive) forms of self-control are restored by pleasant or aroused affect, but such a pattern seems to also challenge the idea of a *shared* self-control resource that would also influence cognitive control.

Another consideration is our particular choices of depletion and self-regulation assessment task combinations. A series of recent studies by Wenzel and colleagues [36] [37] have found that positive moods benefit self-control performance when the depletion task and self-regulation assessment task are different (i.e. resisting candy vs. Stroop), but moods have no effect in the less demanding context with two of the same tasks (i.e. two Stroop tasks). Instead of drawing upon the strength model of self-control to explain this ‘task-switching’ effect, the authors propose an explanation derived from conflict-monitoring theory [38]. Conflict-monitoring theory holds that two systems are involved in solving response conflicts: a conflict-monitoring system and a regulatory system. The conflict-monitoring system detects conflicting stimuli (such as incongruent stimuli on the Stroop task) and the regulatory system resolves the conflict and enables the selection of the appropriate response. Wenzel and colleagues propose that the conflict-monitoring system is impaired when the required response in the depletion tasks conflicts with the required response in the self-regulation assessment (the task switching cost). In such cases positive mood enhances performance because positive mood promotes cognitive flexibility (e.g., [39]). Notably, two experiments [36] [37] found this effect of positive affect on self-control using a Stroop task. These findings suggest that our failure to find an effect of positive mood on self-regulation cannot entirely be attributed to using the Stroop task instead of behavioral measures of self-control.

Wenzel et al [36] have also suggested that the ‘task switching’ effect could explain the findings by Tice et al [2] because all four studies used dissimilar pairs of depletion and self-control assessment tasks. Although our experiments were not designed to test a task switching effect, it is interesting to note that our depletion tasks were also consistently dissimilar, and yet we did not observe an effect of positive affect. In this way, our results also diverge from Wenzel et al.

We cannot be certain why our efforts to find an effect of positive affect on restoration (or cognitive flexibility) were not successful. However, our results should be interpreted within the current context of the ego-depletion literature. Recently a debate has emerged as to whether the ego-depletion phenomenon exists at all. One meta-analysis [18] of 198 published studies on the ego-depletion effect concluded that the effect is a robust finding. However, when reanalyzed using the PET/PEESE statistical methods to compensate for small study effects, such as publication bias, the depletion effect was approximately equal to zero [7]. Other work has revealed problems with the PET/PEESE method, and estimated an attenuated—yet still moderately sized—effect for depletion tasks when using the alternative p-curve method of bias correction [40]. Vigorous debate on the true effect size continues. In studies 2 and 4 (the only studies including repeated Stroop assessments) we observed substantial improvements in Stroop performance after a 5-minute delay. Although practice effects may account for some improvement, it seems likely that a short break (i.e., during mood inductions) was the primary cause of better performance. Examining the mean RTs across trials, over time, for both tasks reveals a stark and substantial improvement from the end of the first task to the beginning of the second task, rather than the continuous gradual improvement a practice effect would produce. In sum, restoration likely occurred, it just did not depend very much or consistently on affective state.

An important consideration in this unresolved depletion debate is that null and inconsistent findings are often not made available to the research community. Therefore, we feel it is important to report null and inconsistent results as openly as possible. We hope that our raw data (osf.io/ax634/) will be of use to those seeking to understand the role of affect in self-regulation and to ego-depletion research, even if no clear picture has emerged across the four studies we conducted on the topic.

### Limitations and Future Research

The present investigation used methods that limit generalizability and conclusions in some potentially important ways. First, we began this research in 2010 with goals to conceptually replicate restoration effects and clarify the potentially distinct roles of valence and arousal. Since then we, like many in Psychology, have learned more about best practices in replication attempts and statistical power. The sample sizes in our research (109, 116, 62, and 59) were generally larger than those collected by Tice et al., [2] (48, 93, 42, and 26); however, it is likely that both sets of studies were underpowered [41]. Future research on this topic would benefit from substantially larger sample sizes.

Second, although we strived to manipulate the valence and arousal of emotions independently, these dimensions are interconnected in complex and subtle ways that may be difficult to separate through self-report measures. Thus, the true independent manipulation of each dimension of emotion is likely difficult, if not impossible, and may not encapsulate real mood states as they exist in daily life. Indeed, the independence between arousal and valence are clear only at the conceptual level; the correlation between these dimensions can vary by personality, timeframe, etc. [42] [43] [44]. Additionally, it is noteworthy that the current investigation did not induce extreme mood states and did not include negative emotions. The broad effect of emotional states, both positive and negative, on restoration remains for future investigation. Future research might also benefit by investigating a wider variety of depletion methods and measures, and include more diverse participant samples.

Third, the number of participants excluded from analyses may limit generalizability of the findings in some ways. Cases were excluded largely for failing to complete the depletion task or not following instructions for completing the Stroop task. It is possible that the relatively monotonous procedures of the four studies also played a role in these instances of failing to comply with instructions; this aspect of the experimental design is itself a potential limitation for generalizability to other contexts. That said, we believe that our conclusions about *these* data are robust across reasonable alternative exclusion criteria, something that could be explored further with our publicly available datasets.

Lastly, because our experimental designs did not include manipulation checks for the depletion task, it is possible that we failed to observe restoration because there was no depletion to restore. Although plausible, this seems unlikely. The ‘crossing out letters’ depletion task used in each of our four studies is commonly used and well validated in depletion research. For example, the same depletion task was found to be effective by Tice et al., [2]. Additionally, Hagger et al.’s [18] meta-analysis of ego depletion included 20 studies (with > 1000 participants) that used the same task in this way and found a substantial average effect size of *d* = .77 (95% CI: .65, .90), and no significant heterogeneity across studies (i.e., non-significant Cochran’s Q). As noted above, our data also suggest a restoration—and thus initial depletion—effect in the two studies that measured Stroop performance over time. Therefore, although we cannot be certain, it seems very likely that our depletion manipulations were successful.

In conclusion, we conducted four studies that investigated the role of affective valence and arousal on restoring cognitive resources following depletion. Our methods included independently manipulating valence and/or arousal (studies 1, 2 & 4), in addition to a more conventional positive mood manipulation (study 3). Across four studies no consistent pattern of results emerged to suggest that affect impacts the rates of cognitive restoration following depletion. Although our manipulations produced substantial differences in affect, the interconnectedness of valence and arousal made independent manipulation somewhat imprecise. In the context of mostly null results, this complexity seems inconsequential. Our findings do not support the conclusions of previous research. Yet, our studies were not highly powered enough to make strong null conclusions. We see the value of this work as prompting questions about the robustness or size of restoration effects, and hope that sharing ambiguous findings will ultimately help move the field towards clarity on hotly debated issues in self-control.
